# Workforce Allocation in Urban Community Mental Health Services: GIS-Based Analytical Insights for Policy and Planning

**DOI:** 10.3390/healthcare13172092

**Published:** 2025-08-22

**Authors:** Somayyeh Azimi, Nasir Uddin

**Affiliations:** 1 Clinical Research Centre, North Metropolitan Health Service, Mental Health, Perth 6010, Australia; nasir.uddin@health.wa.gov.au; 2 School of Health and Clinical Sciences, The University of Western Australia, Perth 6009, Australia; 3 School of Population and Global Health, The University of Western Australia, Perth 6009, Australia

**Keywords:** workforce disparities, health equity, service accessibility Australia, mental health services, health service research

## Abstract

**Background/Objectives:** This study aims to provide a comprehensive understanding of the current mental health workforce and the factors influencing its distribution within adult community mental health services in Western Australia’s North Metropolitan Health Service. **Methods:** Mental health workforce supply across North Metropolitan Statistical Area Level 2 (SA2-Australian Statistical Geography Standard) was estimated using the Geographically-adjusted Index of Relative Supply (GIRS) and categorised as low (0–3) or moderate-to-high (4–8) for analysis and testing associations with multiple covariates. Population, clinic, and individual-level data were analysed using principal component analysis and logistic regression to identify the factors associated with workforce distribution. **Results:** Of the 68 SA2s analysed, 25 SA2s (representing 45 suburbs) were identified as having a low workforce supply, defined by a GIRS score of ≤3. These areas were compared to those with a moderate-to-high supply (GIRS > 3) to assess the differences in service performance. A principal component analysis identified three key components within the data: service usage, health service providers, and service efficiency. A logistic regression analysis revealed that areas with a low workforce supply were significantly more likely to experience reduced service usage (OR = 3.3, *p* = 0.037, CI [0.09–0.92]), indicating fewer patient interactions and lower engagement with mental health services. In addition, these areas demonstrated a lower service efficiency as evidenced by longer wait times (OR = 3.7, *p* = 0.002, CI [1.62–8.50]), suggesting that workforce shortages directly impact timely access to health care. **Conclusions:** The findings revealed disparities in workforce supply across different urban locations, with low-supply areas facing tangible challenges in service accessibility and operational efficiency. These findings highlight the need for targeted mental health workforce planning. Developing and implementing best practice guidelines is essential to effectively manage service demands and reduce waitlists.

## 1. Introduction

The timely provision of mental health (MH) care relies on the continuous and synergistic interaction of multiple factors, including adequate funding and resource allocation, effective coordination among healthcare professionals, accurate diagnosis and treatment, and the accessibility of services. In our previous study, we identified physical distance as a significant barrier to timely care [1]. This study extends that research by focusing on staff resourcing as a critical determinant of timely MH care service delivery. Recent evidence indicates that the current MH workforce is insufficient to meet the needs of Australia’s population, particularly among individuals experiencing moderate-to-severe MH conditions [2,3].

Despite the importance of workforce planning, comprehensive data on the current and recommended staffing levels for MH disciplines in the Australian public sector remains scarce [2]. A recent review has indicated significant gaps in specialised psychiatrist and MH nurse positions. In some States, approximately 25% of psychiatrist positions remain unfilled, resulting in substantial staffing deficiencies within the public MH care services [3]. A 2023 survey by the Royal Australian and New Zealand College of Psychiatrists (RANZCP) revealed that 70% of the respondents experiencing burnout symptoms attributed to workforce shortages [4]. The maldistribution of the workforce further exacerbates these issues, despite a rise in total full-time equivalent numbers [3]. Nurses and psychologists comprise the majority of the specialist mental health workforce in Australia, with around 25,000 nurses and 33,000 psychologists [5]. Nevertheless, Health Workforce Australia (HWA) has projected a 61% gap in the MH nursing workforce by 2030 [6].

Areas with a higher prevalence of MH conditions are more likely to face workforce shortages due to frequent client visits and increased healthcare needs [3,7]. This shortage significantly impacts psychiatric patient care by exacerbating access issues and reducing the quality of care provided. A survey by the RANZCP revealed that more than 90% of the respondents believed that workforce shortages were adversely affecting patient care [4]. A longitudinal study in the US showed that a higher population-based MH staffing ratio was the strongest and most consistent predictor of better treatment quality, access, continuity, and satisfaction [8].

Despite increased government investment in MH care services, many individuals still encounter significant delays in receiving timely care [9]. Insufficient numbers of MH professionals in community settings can lead to increased caseloads per provider, resulting in extended waiting periods for patients seeking care [4] and access to MH care services [8]. A recent Australian study with data from the Medicare Benefits Schedule reported an increase in outpatient wait times for psychiatric appointments. The mean wait time rose from 51 days in 2011 to 77 days in 2025, while the median wait time increased from 15 days to 50 days [10].

Previous studies have demonstrated that access to MH care services is a multifaceted concept, encompassing both realised access (actual service utilisation) and potential access (service distribution and provision) [11]. Geospatial analysis has been identified as a valuable tool for visualising the spatial distribution of health professionals and assisting in service planning and delivery [12]. However, gaps in the data hinder effective workforce planning and the implementation of evidence-based staffing ratios that are crucial for improving health outcomes [2]. Considering this, clinical stakeholders have recommended enhancing national and local health workforce modelling data by investing in advanced business and clinical analysis capabilities [3].

While previous studies have primarily focused on rural workforce disparities, this study is among the first to apply the Geographically-adjusted Index of Relative Supply (GIRS) to assess workforce distribution in urban community mental health settings. This study aims to provide (a) a comprehensive assessment of the current MH workforce supply in the North Metropolitan Health Service (NMHS) area, and (b) examine the factors associated with its distribution across urban areas. Based on the prior research, we hypothesised that disparities in workforce supply may be influenced by factors related to service demand, mental health service accessibility, and broader system-level factors, including the number of consumers, frequency of clinic visits, prevalence of psychosocial impairment, wait times, population demographics, socioeconomic status, and the availability of other health service providers [1,4,13,14]. By integrating the GIRS with multidimensional and spatial data, this study offers a novel approach to identifying the patterns of workforce inequity in a metropolitan context.

This study specifically focused on nursing and allied health staff in adult Community Mental Health (CMH) services in the North Metropolitan Perth area of Western Australia (WA), reflecting their central role in delivering accessible and cost-effective care. Studies have demonstrated that intensive case management (ICM) models, where nursing staff and specialist preventive care clinicians, such as allied health staff, play a pivotal role, lead to significant cost reductions [15,16]. Consequently, WA’s MH care system has transitioned towards community-based care, reducing dependence on hospital-based [15] treatment. This shift aligns with national and state policies that prioritise early intervention and recovery-focused treatment. Despite this shift, there remains a lack of comprehensive information on CMH service provision in WA. This study aims to address the gap by offering evidence-based insights to optimise workforce planning and resource allocation, and to support policy development.

## 2. Materials and Methods

### 2.1. Study Setting

The North Metropolitan Health Service covers 942.4 square kilometres and is home to 703,275 residents as of 2023 [17]. The adult CMH service is a state-funded health initiative and operates community clinics across five distinct areas, each covering specific catchment regions that include a total of 113 suburbs. Within these clinics, teams such as the Assessment and Treatment Team, the Continuing Treatment Team, and the Intensive Clinical Outreach Team provide mental health consultations (in-person, video, telephone), urgent and non-urgent assessments, and comprehensive community rehabilitation services [18].

Each clinic is staffed by a multidisciplinary team including medical, nursing, and allied health professionals. MH nursing roles include registered nurses who practice independently, clinical nurses in senior leadership positions with advanced clinical expertise, and nurse practitioners who are advanced autonomous practitioners [19,20]. Allied health professionals include clinical psychologists, social workers, and occupational therapists. Supplementary Figure S1 provides a schematic model of the intake process in CMH clinics.

Full-time equivalent (FTE) staff data on MH nursing and allied health staff for each of the clinical teams were extracted from WA health active employee’s database and were grouped based on professional level, position, and pay rate, as recorded in WA Health’s active employees’ system.

### 2.2. Study Population and Data Source

In this retrospective cohort study, data were extracted from the Mental Health Information Data (MIND) system—a centralised database of public community mental health episodes and services in WA—for all people aged 18–65 years who received any adult CMH services between January and December 2023 and resided in suburbs within the North Metropolitan Health Service catchment area.

Cases with missing or invalid residential address information were excluded during data processing using Power BI’s data transformation functions. Specifically, records were removed if the suburb field, sourced from the MIND, was either blank or could not be linked to a defined suburb within the Perth North Metropolitan region. This ensured that the analysis was restricted to cases with verifiable geographic information relevant to the study area.

Data extracted included socio-demographic information, diagnoses based on ICD-10 AM and National Outcomes and Casemix Collection (NOCC) measures—a set of standardised tools assessing the clinical status and functioning of MH service consumers at various points in their care.

The Index of Relative Socio-Economic Disadvantage (IRSD) from the Socioeconomic Indexes for Areas (SEIFA), along with the number of GPs and psychologists per Statistical Area Level 2 (SA2), were obtained from the Australian Bureau of Statistics (ABS) census data and the Health Workforce Data [21,22].

### 2.3. Measures

#### 2.3.1. Clinic Measures

Consumer characteristics: Number of adult consumers, number of visits, psychosocial impairment status, gender, and Aboriginal background were used as consumer characteristic variables. Table 1 presents a complete list and description of the clinical variables used in this study. The psychosocial status was assigned based on two factors: (a) an above-average score on two NOCC measures, Life Skills Profile (LSP-16) and Health of the Nation Outcome Scales (HoNOS); and (b) a principal ICD-10 diagnosis of any of the following diagnoses F20, F20.9, F25, F25.9, F30, F41.9, and F99. Further details can be found in our previous study [1].

Waiting time: To assess waiting times for CMH services, we used the following measures:

(a) Waiting time from referral to first service (First service waiting time)—This refers to the average days from the date when a health care provider formally accepts a referral to the first face-to-face service contact excluding the triage event. The metric used for measuring the first service waiting time is “Service Contact Start Date”—“Referral Date.”
(b) Waiting Time from activation to first treatment (First treatment waiting time)—This measure refers to the number of days from activation in a MH care community service to the first service contact within a treatment episode, based on the identified treatment service contacts in community services. The treatment times are calculated as the average across all service units within a community program. The treatment items that include counselling, crisis intervention, depot injection, drug and alcohol rehab/detox, liaison GP, medication review, medication administering, RTMS, and therapy. This wait-time aligns with the Australian Institute of Health and Wellbeing (AIHW) metadata definition of “treatment commencement date”, which is used as a KPI in community MH care.

#### 2.3.2. Population Measures

Demographic and Health Professionals: The number of adults, Aboriginal adults, Non-English-Speaking Background, SEIFA Decile, and the availability of GPs and psychologists by Statistical Area Level 2 (SA2) were collected from multiple sources, including the ABS, LGA records, and NMHS databases. Studies suggested that these demographic variables have strong relationships with workforce supply due to the demand for CMH services and workforce distribution dynamics, such as low SES areas often struggle to retain health care workers, i.e., areas with a higher level of deprivation tend to have fewer GPs and other health professionals [13,14].

#### 2.3.3. Outcome Measures

Mental health workforce supply index: MH nursing and allied health workforce supply in the NMHS area was estimated using the GIRS from AIHW [14]. The GIRS adjusts the known MH workforce supply in an area based on four components, including workforce rate, land size, population dispersion, and the approximate travel time [13,14]. Each of the four components is assigned an integer value between 0 and 2, with zero suggesting the greatest challenge. This study used a modified GIRS score which includes the following: i MH workforce rate, which represents the availability of nursing and allied health professionals. This was determined by the FTE rates per 100,000 adult population (aged 18 to 65 years) based on 2023 WA population estimates. Children under 18 years and adults over 65 years were excluded due to the clinical services examined in this study. The workforce rate was calculated using the following formula: Workforce Rate = (FTE by clinics/Adult population (18–65) in SA2 that are within the clinic catchment area) × 100,000. This measure standardises the workforce availability, enabling comparisons across different regions regardless of population size. The FTE rate is excluded from calculations for SA2 where the adult population is ≤500 or where population density is ≤1 person per km^2^. These exclusions ensured the reliability of the workforce rate by avoiding distortions caused by extremely small populations or sparsely populated areas. ii. Land size of each SA2, which is measured in square kilometres (km^2^) and extracted from the 2021 ABS data. It provided a geographical measure of the area being analysed. iii. Population density, which is calculated as the adult population of SA2 divided by the land area (population/km^2^) and indicates how dispersed or concentrated the population is within a given SA2. iv. Median distances were used as a proxy for approximate travel time between the consumers’ residential suburbs and the nearest community mental health (CMH) clinics. Distances were calculated in Power BI using the Haversine formula, implemented with Data Analysis Expressions (DAX). The formula estimates the great-circle distance between two points on the Earth’s surface from their latitude and longitude coordinates, expressed in kilometres as the shortest possible path rather than the road network distance. Coordinates were obtained for each of the five CMH clinics and the central points of residential suburbs linked to Statistical Area Level 2 (SA2).

The four components were then added together to obtain the total GIRS score, ranging from 0 to 8. A score of 0 indicates areas with significant challenges in the MH workforce supply, characterised by large, sparsely populated areas, and in the bottom 25th percentile of the FTE rates calculated, with a greater distance from CMH clinics. Conversely, a score of 8 indicates small, densely populated areas, ranked in the top 25th percentile of the calculated FTE rates with a lesser distance to MH care services. For a detailed explanation of the scoring method for the four GIRS components, see Supplementary Table S1. The GIRS score was further binarised into low (0–3) and moderate-to-high (4–8) MH workforce supply [13] for the analysis and testing associations with multiple predictors. Based on Hill et al. 2023 [13], a GIRS score ≤ 3 reflects a critical shortage.

### 2.4. Statistical Analysis

All data were stored in Excel spreadsheets and processed using Power BI (Version: 2.122.1066.0 64-bit-October 2023) to integrate various data segments and sources for analysis. Jamovi open-source software (version 2.2.5.0) was used for the data analyses. Descriptive statistics and normality testing were performed to understand the key characteristics of the data dispersion and distribution shape. This helped to identify outliers of the study variables and to limit extreme values in non-normally distributed data. The FTE rate per one thousand consumers in each clinic and the GIRS score were reported against the SA2 boundaries as indicators of MH workforce supply. Additionally, average first service waiting time and first treatment waiting time were calculated by extracting data from MIND and SQL programming.

The spatial distribution of MH nurses and the allied health workforce supply, based on the GIRS score by suburbs and frequency of visits, was visualised using Power BI.

This study is designed as exploratory research, as there was not enough evidence in the prior literature about the application of some of the key metrics related to service factors, such as the waiting times for CMH services. Moreover, there were several population, clinical, and demographic variables with high collinearity and dimensionality issues. The principal component analysis (PCA) is a dimensionality reduction technique that transforms correlated variables into a set of components that capture the maximum variance in the data. This approach allowed us to simplify the model while retaining the most significant information from the original variables. ProMax rotation was selected to account for the expected correlations between components, as the underlying factors influencing workforce distribution were assumed to be interrelated rather than orthogonal. Following the PCA, binominal logistic regression was used to test the hypotheses related to the association between the predictor variables and the workforce supply. This study operationalised the ‘Workforce Supply’ as an independent binary outcome variable with two categories, ‘1 = low supply’ and ‘0 = moderate-to-high supply’ areas, as described in the section on the workforce supply index.

The reporting follows the Strengthening the Reporting of Observational Studies in Epidemiology (STROBE) statement. An exemption from the Human Research Ethics Committee review was obtained from the North Metropolitan Area Health Service MH Human Research Ethics Committee (NMHSMH HREC) after this study was approved as a quality improvement activity (GEKO: 52492).

## 3. Results

Across five adult CMH clinics in NMHS, a total of 121.50 FTE mental health nursing and allied health staff were employed, including clinical psychologists, social workers, occupational therapists, and nurses (Table 2 and Table S2). Among nurses, registered nurse represented the most junior pay category, with a 2.4 FTE, compared with 41.42 FTE clinical nurses and 20.93 FTE nurse specialists, indicating that clinics were primarily staffed by experienced clinicians. Clinic-A had the highest number of FTEs (33.8), while Clinic-C had the highest FTE rate per 1000 consumers (38.7). Conversely, Clinic-E had the lowest number of FTEs (15.8) but the second-highest FTE rate per 1000 consumers (37.3). This combination of relative staffing and demand of service may indicate underfunding or resource misalignment.

**Figure 1:**
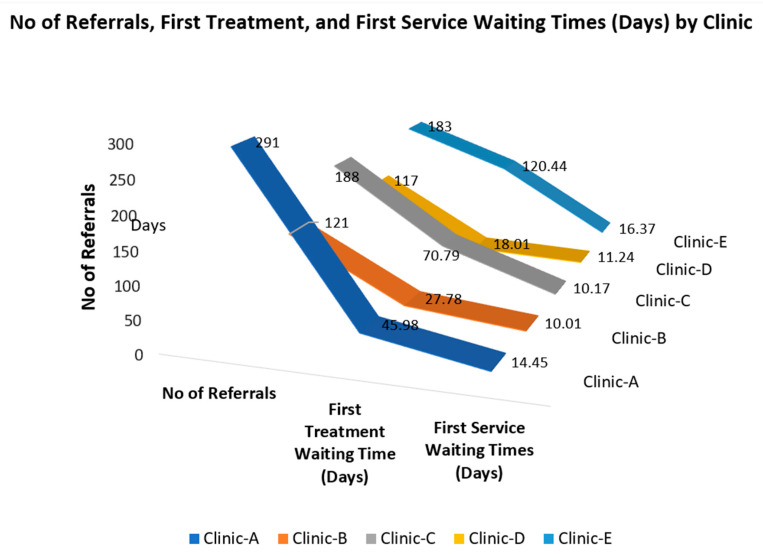
Number of referrals, first treatment, and first service waiting times by each clinic.

Over the period of 2023, a total of 4008 adult consumers made 70,189 visits to the adult CMH clinics’ services within NMHS-MH. Supplementary Table S3 shows demographic characteristics of consumers who received services from each of the five clinics. In Figure 1, the Y-axis represents the number of referrals, while the X-axis displays three categorical metrics, including number of referrals, first treatment waiting time, and first service waiting. Each line represents a clinic, allowing for a visual comparison across the three metrics. Parallel trends across clinics suggest similar patterns in service delivery, while divergence between the lines indicates differences in service access or efficiency.

**Figure 2:**
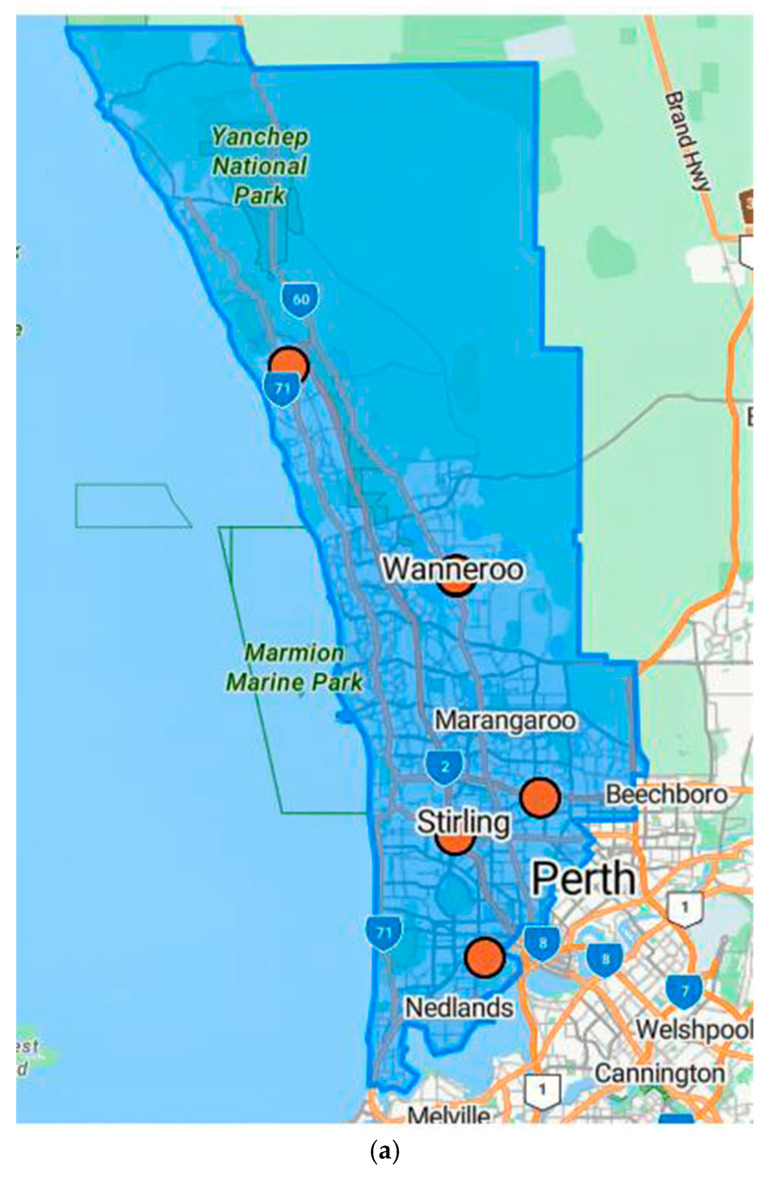

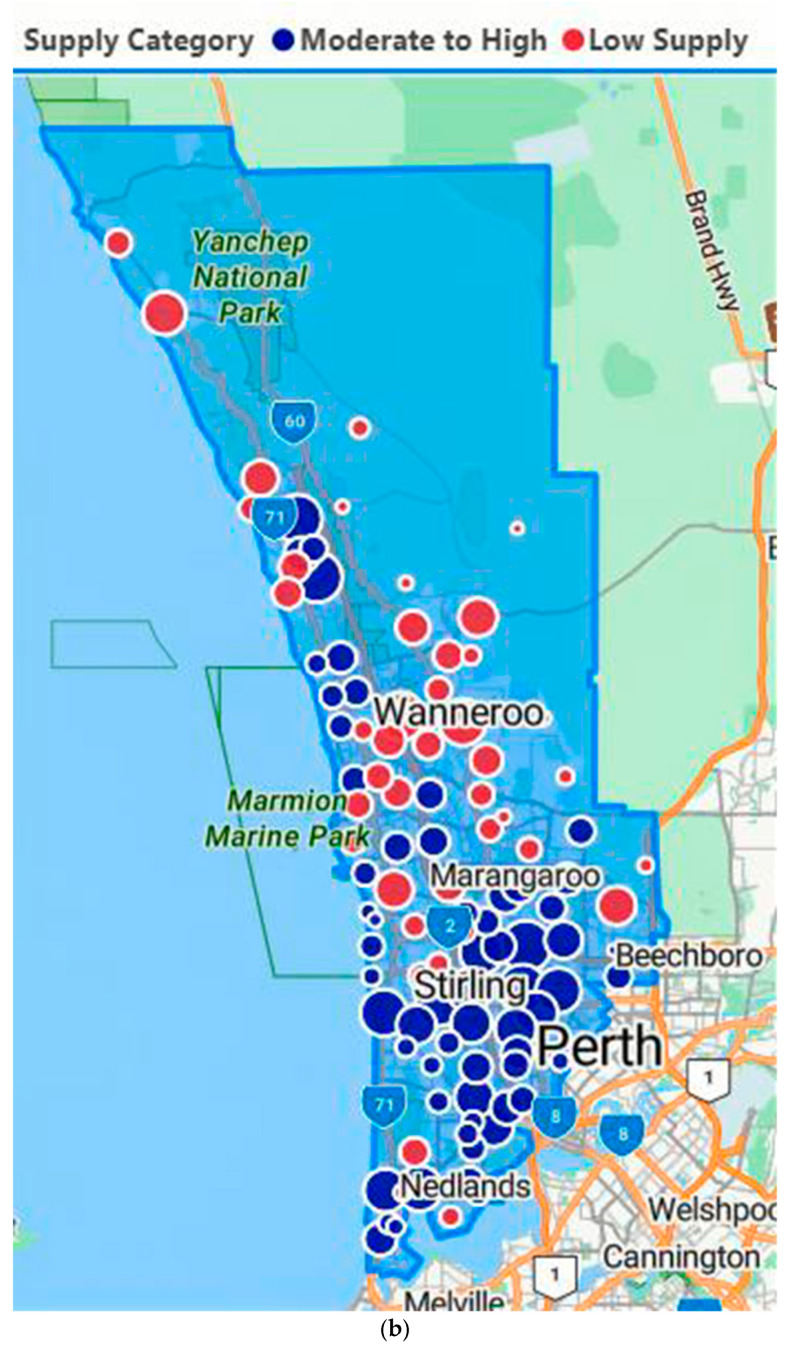
(**a**) Clinics’ location “Orange Circle”; (**b**) spatial distribution of workforce supply based on the GIRS score with overlayed adult consumer visits in North Metropolitan Suburbs. “Red = low supply, Blue = Moderate-high supply”—Blue line shows North Metropolitan Perth Health Service boundaries. The numbers refer to the road network and are not related to the study data.

A total of 25 SA2 areas that represents 45 suburbs within the NM catchment areas were identified as low-supply spots based on a GIRS score ≤ 3. Figure 2 illustrates the clinics’ location and the spatial distribution of workforce supply with overlayed client visits and suburbs linked to the respective SA2 in NMHS boundaries.

To evaluate the association between the MH workforce supply distribution and other predictors, a PCA analysis was conducted. Variables that had a uniqueness value greater than 0.5 were removed, ensuring that only those contributing meaningfully variables were retained for further analysis. From the PCA, three main structured components were identified from the data (Eigenvalue > 1, cumulative variance = 82.3%, KMO = 0.74, Bartlett’s Test *p* < 0.001). Table 3 shows the correlations (loadings) between individual variables and the principal components. Component 1, representing “Service Usage”, is strongly associated with adult consumers, adult visits, female consumers, consumers with psychosocial disabilities, and Aboriginal population and Aboriginal consumers. Component 2, representing “Healthcare Availability & SES”, includes the number of GPs, psychologists, and the SEIFA decile. Component 3, representing “Service Efficiency”, includes first service and first treatment waiting times. The three components collectively explained 82.2% of the variance, with ‘Service Usage’ accounting for the largest share (47%).

We conducted binary logistic regression to estimate the probability of associations between workforce supply (1 = low, 0 = moderate-to-high) and the predictor variables identified through the PCA analysis. Two models were built for this analysis, setting ‘moderate-to-high’ as the reference category to estimate the likelihood of associations with low workforce supply. In the first model we included only the components derived from the PCA analysis. In the second model, building on our previous findings [1] that linked disadvantaged SEIFA areas to higher service attendance rates as well as increased psychological distress and psychosis, we added the SEIFA decile, the Aboriginal population count, and their interaction term to explore SES disparities in Indigenous communities. We were particularly interested in examining whether the SES influence workforce supply distribution in areas with varying sizes of Aboriginal populations, as areas with larger Aboriginal populations may face distinct workforce challenges depending on their SES. Table 4 presents the regression results (Sample size = 68 SA2). The results indicated that low workforce supply was strongly associated with lower service usage and high wait times as operationalised for service efficiency. However, no association was found between health care availability and workforce supply; saturation in the private sector may compensate for the shortages in the public system. In Model 1, Table 4, the analysis indicates that service usage and service efficiency (OR = 3.72, *p* = 0.002, 95%CI [1.62–8.50]) had significant predictive power associated with low workforce supply and that, when the predictors increase by one unit, the odds of the outcome occurring increased by approximately three times. Model 2, Table 4 expands on this by adjusting for SES and the Aboriginal population, by adding these as additional predictors; the interaction between those (Aboriginal population X SEIFA Decile) showed a nuanced impact on workforce supply (χ^2^ (3) = 3.99, *p* = 0.26). Model 2 demonstrated better fit (McFadden’s R^2^ = 0.38; R^2^N = 0.54; AIC = 52.9; Deviance = 38.9) compared to Model 1 (McFadden’s R^2^ = 0.32; R^2^N = 0.46; AIC = 50.9; Deviance = 42.9), indicating improved explanatory power despite a slight increase in model complexity, and offering a more comprehensive understanding of workforce supply dynamics.

**Figure 3:**
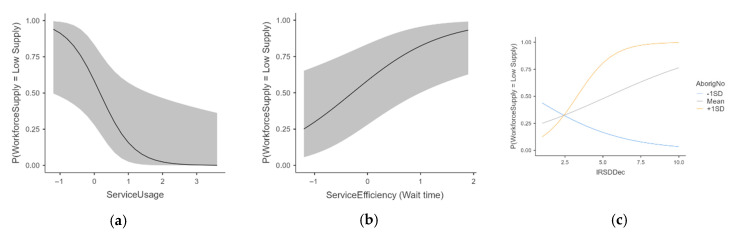
(**a**–**c**): Estimated marginal mean for significant variables for (**a**): service usage, (**b**): service efficacy, and (**c**) interaction of Aboriginal status and SES-Decile. The grey area is 95% confidence interval.

Further to the results, we calculated the estimated marginal means to represent the predicted probability of low workforce supply at specific levels of the predictor variables, holding other variables constant. This has allowed for a clearer understanding of the practical implications of the model, as presented in Figure 3a–c. In Figure 3a,b, the X-axis represents the predictors with a standardised mean value ranging from −1 to 3, while the Y-axis represents probability from 0 to 1. This demonstrate a strong association, indicating that areas with low workforce supply are closely linked to low service usage and/or high wait times. In Figure 3c, although the interaction term is not significant, the mean trend line represents how strongly Aboriginality contributes in reference to the odds of association with low workforce supply areas.

## 4. Discussion

### 4.1. Principal Findings

The findings revealed substantial gaps in workforce supply across different suburbs of the NMHS CMH, as indicated by the GIRS ranging from 0 (most deprived) to 8 (most affluent). A logistic regression analysis confirmed that low workforce supply is strongly associated with low service usage and significantly reduces service efficiency, as evidenced by the high wait times.

### 4.2. Comparison with Previous Studies

The previous literature has highlighted the shortage of the MH workforce in Australia [3,23]; however, no prior study has used the GIRS to examine the factors influencing CMH workforce shortages. Given the crucial role of CMH in reducing hospital readmissions [1,24], understanding these factors is essential for effective service planning and policy development.

The regression analysis indicates that areas with lower workforce tend to serve fewer service users, potentially indicating lower community engagement with the MH care system. These findings are consistent with prior studies that highlight the role of workforce availability in supporting patient engagement and service utilisation [25]. Limited engagement in areas with insufficient workforce capacity may lead to unmet MH needs and poorer outcomes, reinforcing the importance of equitable workforce distribution to improve service delivery.

There is a disparity between the demand for MH care services and the available workforce in Australia. Nearly one in six West Australian adults reported experiencing a MH condition in the past 12 months [26,27]. Among those who reported needing professional support, 38% experienced delays or missed at least one scheduled appointment [28]. Meanwhile, the MH workforce has shortages across various occupations and disciplines [29], with a projected national shortfall of 18,500 MH nurses by 2030 [23,30]. To address this, the Mental Health Clinical Workforce Action Plan identified five priority action areas, including reviewing and reforming, training, attracting and retaining, maximising capability, and supporting workforce wellbeing [27].

Attracting and retaining MH professionals, as well as ensuring an appropriate distribution and skill mix to meet both current and future population needs, remains a significant challenge [23], partly due to the availability of more profitable and less stigmatising career options [31]. Recent research on the Australian Community MH Workforce [32] has revealed that a significant portion of staff identified recruitment, retention, and skill level gaps as major challenges in delivering the Commonwealth Psychosocial Support Program. Staff mentioned short-term contracts, burnout, and job insecurity as major retention issues. Immediate action is needed, as almost half of them expressed job dissatisfaction and an intention to leave [32].

One innovative strategy to improve clinical outcomes and to expand service availability is redistribution of the workforce from higher supply to those with limited supply. This approach aims to balance the GIRS scores across areas, ensuring more equitable access, especially for individuals with psychosocial disabilities. Improving competencies, redefining roles and responsibilities, and adopting innovative team structures can also contribute to a more balanced skill set and better workforce distribution [23,33].

The finding also revealed that, in low workforce areas, waiting times were over three times longer, indicating reduced service efficiency. Therefore, unlike in rural settings, urban workforce shortages are more closely associated with wait times than with geographic distance [12,34]. Waiting times for health services often indicate a system under strain [35], and addressing these disparities requires targeted interventions [32]. Efficient service delivery is vital for maintaining patient engagement and ensuring timely access to care [36].

While waiting time is commonly defined as the median duration from referral to service activation date [35], this study measured the waiting time from referral to first service contact date to align with the NMHS-MH key performance indicators (KPIs). Also, average waiting times were used instead of median waiting times. While the median may better reflect policy changes—given that waiting times for CMH services are typically right-skewed, with many short waits and fewer long waits—this study uses the average waiting time per clinical unit to assess the system-wide impact and its relationship with workforce supply.

Extended waiting times and treatment delays in mental health care can reduce patient satisfaction and worsen clinical outcomes [36]. It can lead to maladaptive coping mechanisms, irrational beliefs, and higher no-show or dropout rates. Additionally, higher no show or “did not attend” (DNA) rates contribute to care delay for others and increase the demand for private services [36]. Poor engagement due to long waits can result in MH deterioration and increased hospital admissions [37].

We did not find any significant correlation between the first service waiting time and the SEIFA decile (Pearson r = 0.02, *p* = 0.885), which is consistent with the previous research [38,39]. Therefore, although it has been argued that lower SES groups are disproportionately affected [35], this relationship needs further investigation.

To mitigate waiting times as a barrier to access to MH care, systematic reporting and innovative health care delivery models are essential. A recent systematic review highlighted various approaches, such as triage processes, patient-led approaches, walk-in clinics, multidisciplinary services, and changes to service delivery models [40].

One effective strategy for reducing waiting times involves modifying triage processes—for example, by standardising and centralising intake procedures and integrating triage with brief assessments [40]. In the UK, the NHS’s Improving Access to Psychological Therapies program introduced national standard targets to reduce waiting times to address long delays in public mental health care [37,41]. In Australia, a randomised trial demonstrated a 34% reduction in waiting time for community outpatient services by combining triage with initial management through the specific timely appointments for triage (STAT) model [19]. Although our CMH model of care uses the standardised crisis triage rating scale (CTRS) for triage process, combining this model with STAT may help reduce waiting times, especially in low workforce areas.

Changes to service delivery, such as brief group intervention models, offers a practical solution in resource-limited areas [40]. However, this approach carries the risk of overburdening clinicians and reducing the quality of care [36].

Other strategies for managing waitlists include conducting systematic audits to ensure accuracy, verifying that individuals on the list are actively waiting for appointments, and offering remote rapid intakes and mental wellness screenings [36]. Additionally, developing e-portals for consumers can help them track their referral progress and access relevant information [42].

Supporting consumers during the waiting period is another crucial strategy [42]. An Australian study reported that almost half of consumers experienced MH decline while waiting for treatment, highlighting the need for supportive lifestyle services during this period, such as exercise, sleep, healthy eating, and psychological assistance [9].

This study found no significant relationship between the number of GPs and psychologists and areas with low workforce supply, possibly due to their concentration in the private sector in the metropolitan area. This suggests that, while the presence of other healthcare providers is important, it may not directly influence MH workforce supply in the public sector.

The findings for the interaction between the SEIFA decile and the Aboriginal population suggest that the strong association between socioeconomic advantage and workforce supply is not uniform across all population settings. Specifically, in areas with a higher population of Aboriginal people, the likelihood of low workforce supply remains elevated even in the socioeconomically least disadvantaged regions (SEIFA deciles 7–10). This pattern aligns with the national evidence and suggests that structural and cultural barriers to service access persist regardless of broader socioeconomic advantage [43].

### 4.3. Strength and Limitation

The strength of this study lies in its use of real-time clinical service data, providing direct insights for policymaking. The collaboration with clinicians ensured the practical relevance of the findings. The use of a standardised AIHW index combined with multi-dimensional predictors at the population, clinic, and individual levels enabled a robust workforce evaluation.

Limitations include a reliance on administrative data. Also, the analysis did not consider DNA rates and engagement with the system, which could affect the interpretation of waiting times. Additionally, the workforce supply was a measure using the FTE counts, which do not reflect clinician experience levels. This means that simply having enough FTE positions does not necessarily ensure efficient service delivery. This study also did not calculate waiting times separately for various diagnoses, which could differ significantly.

Both this study and our previous report examined service data from a single Perth Metropolitan MH care service area. Extending the scope to the entire metropolitan region would yield broader implications for mental health care coordination within a modern urban agglomeration.

### 4.4. Implications for Practice and Policy

One key implication of this study is the necessity to balance workforce distribution according to population density and workforce availability to ensure more equitable access to services. This approach is particularly crucial for vulnerable service users, such as individuals with psychosocial disabilities and Aboriginal communities. Achieving this requires clear implementation plans along with ongoing evaluation and monitoring to ensure effectiveness and impact.

To address the critical issue of waiting times and to improve service efficiency, transparent reporting, streamlined data collection, and targeted interventions are crucial. Using innovative methods, such as telehealth or the STAT model, can help reduce waiting times.

Developing best practice guidelines for managing demand and active waitlists is essential to equip the CMH services with practical tools and information to optimise resource use.

### 4.5. Future Research

Future studies should broaden the geographic scope to include both metropolitan and rural areas and to incorporate qualitative insights from clinicians and service users to deepen contextual understanding. Pairing GIS analysis with interviews in a mixed-methods or longitudinal design could offer richer explanations of workforce patterns. In addition, stratifying wait times by diagnosis and urgency, and considering staff competencies may better inform equitable and effective workforce planning.

## 5. Conclusions

This study is the first to provide a comprehensive evaluation of the adult CMH services workforce in a metropolitan area in WA. The findings revealed disparities in workforce supply across different locations using the standard GIRS. It was observed that workforce supply can have a significant impact on service usage and efficiency. This study recommends adopting novel service delivery methods, prioritising recruitment in low-GIRS suburbs via retention incentives, and leveraging technology to support consumers during their waiting periods, which will enhance resource optimisation in MH care community services.

## Figures and Tables

**Table 1:** Full list of variables and definitions.

Variable Name	Definition
Adult consumer numbers	Number of individuals (18–65 years old) receiving any type of care (face-to-face, phone consultation, or video conference) from each community mental health clinic in North Metropolitan Perth.
Adult consumer visits	The cumulative number of service events where individuals (18–65 years old) received care from a community mental health clinic, provided in various venues. Each consumer can have multiple visits, encompassing all interactions with the clinic.
Consumers with psychosocial impairment	The psychosocial status was assigned for a consumer based on two factors: (a) an above-average score on two NOCC measures, Life Skills Profile (LSP-16) and Health of the Nation Outcome Scales (HoNOS), and (b) a principal ICD-10 diagnosis of any of the following diagnoses F20, F20.9, F25, F25.9, F30, F41.9, and F99.
First service waiting time	Average days to the first face-to-face service contact, excluding the triage event, from the date when a health care provider formally accepts a referral.
First treatment waiting time	Average days from activation in a MH care community service to the first service contact within a treatment episode, based on the identified treatment service contacts in community services.
Population demographic	Number of adults, Aboriginal adults, Non-English-Speaking Background (NESB) by Statistical Area Level 2 (SA2).
Socio-Economic Indexes for Areas (SEIFA) Decile	Set of indexes developed by the Australian Bureau of Statistics (ABS) to rank areas in Australia according to their relative socio-economic advantage and disadvantage: Decile 1: Most disadvantaged. Decile 10: Least disadvantaged.
Health Professionals	Number of GPs and psychologists by SA2.

**Table 2:** Distribution of mental health staff across five community clinics based on FTE rates (total count and per 1000 consumers).

	FTE/Clinics	FTE Community Clinics					FTE/1000 Consumers Community Clinics				
Professions		A	B	C	D	E	A	B	C	D	E
Clinical Nurse Specialist		4.8	6.0	2.8	4.4	3.0	3.9	5.7	4.8	6.0	7.1
Clinical Psychologists		2.9	1.7	1.9	1.3	1.0	2.4	2.2	3.7	2.5	2.8
Registered Nurse		14.5	8.7	6.4	8.0	6.2	12.0	8.2	11.0	11.0	14.7
Occupational Therapist		5.6	5.4	5.0	6.4	3.0	4.6	5.2	8.6	6.4	7.0
Social Worker		6.0	5.0	6.5	4.1	2.6	5.0	4.7	11.1	5.6	6.2
Total		33.8	26.8	22.7	22.5	15.8	27.8	25.4	38.7	30.8	37.3

**Table 3:** Principal component analysis (PCA) of the full data set (N = 68).

	**PCA Components**	**PCA 1: (47%)** ***Service Usage***	**PCA 2: (18.9%)** ***Healthcare Availability & SES***	**PCA: (16.3%)** ***Service Efficiency***
**(% of Variance)** **Variables**				
Adult consumers		0.99		
Adult visits		0.97		
Female consumers		0.96		
Psychosocial disability consumers		0.95		
Aboriginal consumers		0.84		
Aboriginal population		0.55		
Clinical psychologists No			0.91	
GP No			0.81	
SEIFA deciles			0.57	
Average treatment time				0.93
Average wait times to first visit				0.91

**Table 4:** Logistic regression analysis results.

(a) Model 1							
Predictor	Estimate	SE	Z	*p*	Odds Ratio	Lower	Upper
**Intercept**	−0.569	0.4	−1.421	0.155	0.566	0.2585	1.241
**Service Usage**	−1.203	0.576	−2.088	0.037	0.3	0.0971	0.929
**Healthcare Availability and SES $**	0.213	0.382	0.558	0.577	1.238	0.5855	2.616
**Service** **Efficiency**	1.314	0.421	3.118	0.002	3.721	1.6289	8.501
**(b) Model 2**							
**Predictor**	**Estimate**	**SE**	**Z**	***p***	**Odds Ratio**	**Lower**	**Upper**
**Intercept**	0.64731	3.12544	0.207	0.836	1.91	0.00418	873.984
**Service** **Usage**	−2.01874	0.92646	−2.179	0.029	0.133	0.02161	0.816
**Healthcare Availability & SES $**	0.51576	0.68067	0.758	0.449	1.675	0.44116	6.359
**Service** **Efficiency**	1.19479	0.46072	2.593	0.01	3.303	1.33882	8.148
**Aboriginal No**	−0.01014	0.00859	−1.18	0.238	0.99	0.97338	1.007
**SEIFA** **Decile**	−0.56851	0.48262	−1.178	0.239	0.566	0.21994	1.458
Aboriginal-No * SEIFA@ Decile	0.00417	0.0023	1.813	0.07	1.004	0.99966	1.009

$ Socio-economic Status, @ Socio-Economic Index for Areas. “*” is a symbol for interaction between the terms “Aboriginal No“ and “SEIFA”.

## Data Availability

Data were extracted from the Mental Health Information Data Collection and Psychiatric Services Online Information System. Due to privacy considerations and to protect the confidentiality of the study participants, the data cannot be openly shared.

## Supplementary Materials

The following supporting information can be downloaded at: https://www.mdpi.com/article/10.3390/healthcare13172092/s1, Figure S1: Schematic model of intake process and the time patients spend waiting for care; Table S1: Assigning Score for each GIRS Component; Table S2: Mental Health Workforce Position by Pay Rate; Table S3: Number of Service Users in Each Clinic.
