# Supplementary material for: Workforce Allocation in Urban Community Mental Health Services: GIS-Based Analytical Insights for Policy and Planning
Source: Healthcare (Basel). 2025 Aug 22;13(17):2092. doi: 10.3390/healthcare13172092 (PMC12428168; doi:10.3390/healthcare13172092)
Supplement: Supplementary file 1 [file healthcare-13-02092-s001.zip › healthcare-3758327-supplementary.pdf]

Workforce Allocation in Urban Community Mental Health Services: GIS-Based Analytical Insights for Policy and Planning

Supplementary tables:

Table S1: Assigning Score for each GIRS Component

Score	Workforce rate	Land size	Population density	Distance to clinic
**0**	Bottom 25%	Top 25%	Bottom 25% density	farthest 25% distance to clinic
**1**	Middle 50%	Middle 50%	Middle 50% density	Middle 50% distance to clinic
**2**	Higher 25%	Smallest 25%	Top 25% density	Nearest 25% distance to clinic

Table S2: Mental Health Workforce Position by Pay Rate

Rate Name	Clinical Nurse specialist	Clinical Psychologist	Clinical Nurse	Occupational Therapist	Registered Nurse	Social Worker
**Clinical Psychologist (6/3)**		1.3				
**Clinical Psychologist (7/3)**		1.5				
**Clinical Psychologist (8/1)**		0.5				
**Clinical Psychologist (8/2)**		0.5				
**Clinical Psychologist (9/1)**		1				
**Clinical Psychologist (9/2)**		4.03				
**Gen DIV L8/1**				0.5		
**Prof DIV L1/1**				0		
**Prof DIV L1/2**				2		1.5
**Prof DIV L1/3**						2
**Prof DIV L1/4**				1.4		2.8
**Prof DIV L1/5**				0.6		
**Prof DIV L1/6**				6.58		5.5
**Prof DIV L2/2**				0		
**Prof DIV L2/3**				12.64		12.4
**REG GEN Nurse L1-3**					0.4	
**REG GEN Nurse L1-8**					2	
**S-DEV/CLIN/A-MAN L2-1**			2.12			
**S-DEV/CLIN/A-MAN L2-2**			0.61			
**S-DEV/CLIN/A-MAN L2-3**			1.27			
**S-DEV/CLIN/A-MAN L2-4**			37.42			
**SENIOR REG NSE L2**	2.42					
**SENIOR REG NSE L3**	17.51					
**SENIOR REG NSE L7**	1					
**Total**	20.93	8.83	41.42	23.72	2.4	24.2

Table S3: Number of Service Users in Each Clinic

Clinic Name	Adult Consumers No	Adult Consumers Visit	Female Patient No	Male Patient No	Aboriginal Adult Consumers	Number consumers with Psychosocial disabilities
**Clinic-A**	1,212	18,640	674	538	50	319
**Clinic-B**	1,057	14,615	525	532	92	487
**Clinic-C**	586	14,829	264	322	21	283
**Clinic-D**	730	12,516	366	364	32	279
**Clinic-E**	423	9,589	229	194	27	174

Supplementary Figures:

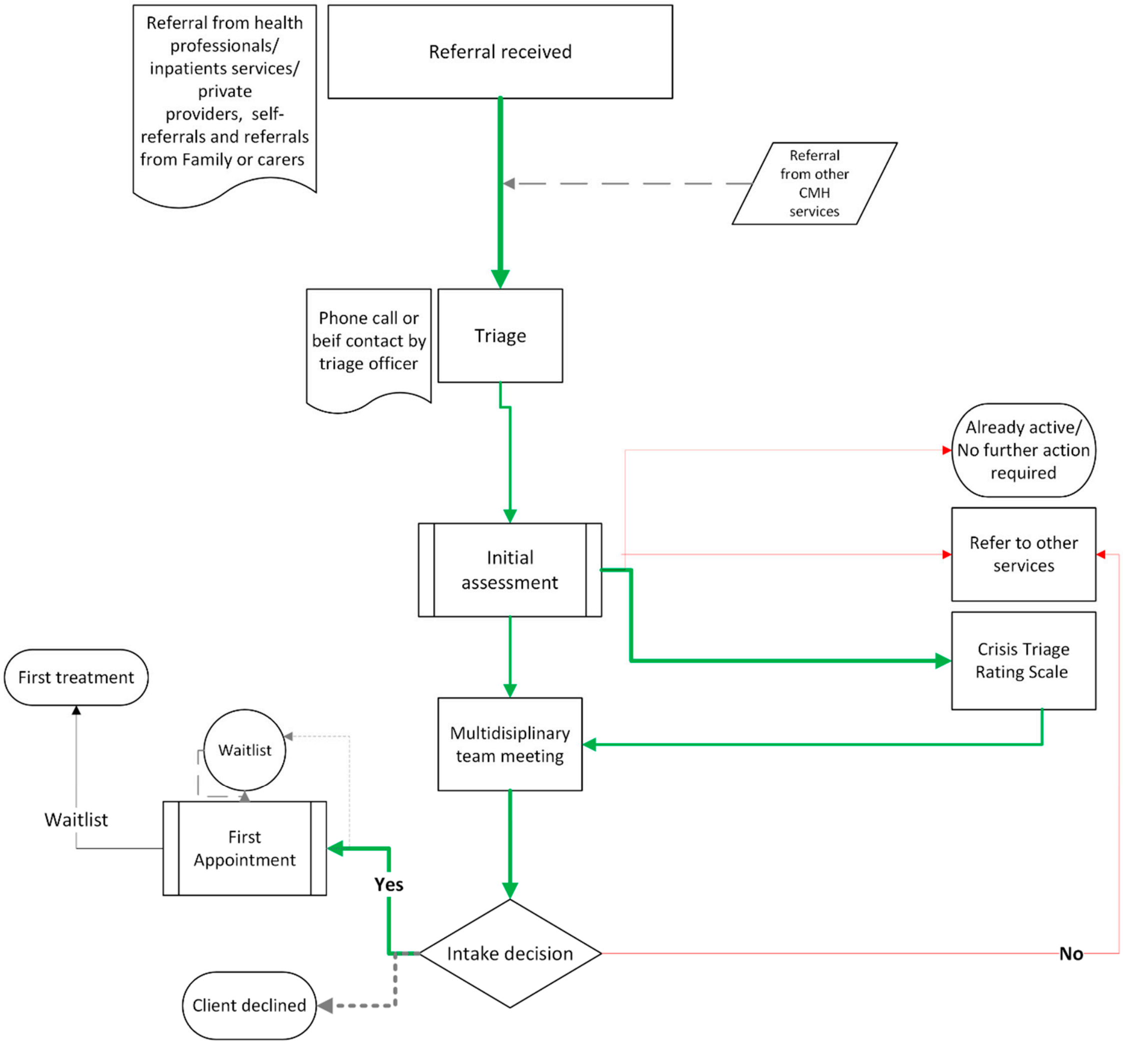

Figure S1: Schematic model of intake process and the time patients spend waiting for care
